# Increased freedom of movement in the nascent chain results in dynamic changes in the structure of the SecM arrest motif

**DOI:** 10.1042/BSR20181246

**Published:** 2019-01-18

**Authors:** Hazel A. Bracken, Cheryl A. Woolhead

**Affiliations:** Institute of Molecular, Cell and Systems Biology, College of Medical, Veterinary and Life Sciences, University of Glasgow, Glasgow G12 8QQ, U.K.

**Keywords:** bacterial secretion, protein synthesis, ribosomes, translation arrest

## Abstract

Ribosomes are responsible for the synthesis of all cellular proteins. Due to the diversity of sequence and properties, it was initially believed that translating nascent chains would travel unhindered through the ribosome exit tunnel, however a small but increasing number of proteins have been identified that interact with the exit tunnel to induce translational arrest, *Escherichia coli* (*E. coli*) secretion monitor (SecM) is one such stalling peptide. How and why these peptides interact with the exit tunnel is not fully understood, however key features required for stalling appear to be an essential peptide arrest motif at the C-terminus and compaction of the nascent chain within the exit tunnel upon stalling. Mutagenesis of the SecM arrest sequence has identified three conservative point mutations that can retain a degree of stalling in this highly conserved sequence. This level of stalling is further increased when coupled with mutation of a non-essential arrest motif residue P153A. Further analysis of these mutants by pegylation assays indicates that this increase in stalling activity during translation is due to the ability of the P153A mutation to reintroduce compaction of the nascent chain within the exit tunnel possibly due to the improved flexibility of the nascent chain provided by the removal of a restrictive proline residue. The data presented here suggest that arrest sequences may be more prevalent and less highly conserved than previously thought, and highlight the significance of the interactions between the nascent chain and the exit tunnel to affecting translation arrest.

## Introduction

For ribosomes to translate thousands of peptides efficiently, non-specific interactions with the ribosome tunnel must be avoided, to prevent slowing the rate of translation. However, translation arrest peptides have been shown to have specific, critical functions for gene expression and monitoring cell homoeostasis. Therefore, these arrest sequences must have sufficient complexity to maintain their specificity. As such the intricate details of how arrest peptides function is still not well understood. Secretion monitor (SecM) is one of the best studied examples, in *Escherichia coli* SecM monitors SecYEG translocon export activity through its own translocation to the periplasm and up-regulates translation of SecA, an ATPase involved in the SecYEG translocation machinery, when translocation is reduced [1]. In recent years, the SecM stalling peptide has also become an essential biochemical tool in creating stable ribosome-bound nascent chain complexes, for studying the timing and process of co-translational folding and targeting [2–4].

The 170-amino acid SecM monitors cell export activity through its own translocation to the periplasm and up-regulates translation of SecA when translocation is reduced. Once exported to the cytoplasm, the function of SecM is complete and it is rapidly degraded by tail-specific proteases [5]. The SecM peptide contains a signal sequence in its N-terminus which co-translationally targets it to the Sec translocation machinery at the cell membrane [6], and an arrest motif in its C-terminus that interacts with the ribosome exit tunnel and induces elongation arrest at Pro^166^ [7]. When the *SecM-SecA* mRNA is initially transcribed the SecA Shine–Dalgarno sequence is sequestered by the mRNA secondary structure. Stalling of the ribosome translating SecM disrupts this mRNA hairpin exposing the Shine–Dalgarno sequence, thus enabling the translation of SecA [8]. Under normal conditions when the secretion status of the cell is sufficient, the stalling of SecM translation is transient, with a half-life of less than 1 min, and the translation of SecA is basal [5]. This transient stalling is released by the ‘pulling’ of the polypeptide through the SecYEG translocation machinery (Butkus et al. (2003) [9], Goldman et al. (2015) [10]). However, when cell secretion is impaired, this release of stalling does not occur and the Shine–Dalgarno sequence of SecA remains exposed, resulting in its up-regulated translation and thus increased production of SecA.

SecM undergoes translation elongation arrest at Pro^166^, four residues prior to the termination point. At this point tRNA-Pro^166^ is situated at the ribosome A-site, however, a peptide bond does not form between Gly^165^ and Pro^166^, therefore the proline separates and is not incorporated into the peptide chain [11]. The presence of the Pro^166^-tRNA in the A-site is, however, still essential for translation arrest. Studies have shown that while the key arrest motif residues are critical for stalling *in vivo*, alterations to the arrest motif can be compensated for through mutation of one or more flanking residues that enable positioning of the critical Arg^163^ residue to form crucial interactions with the ribosomal exit tunnel that are required to induce stalling (Yap and Bernstein (2009) [12]). This was supported by molecular dynamics flexible fitting (MDFF) modelling data, which showed it is the key interaction of R163 with the rRNA nucleotide A2062 that is essential for stalling. This interaction results in a potential relay communication between the SecM nascent chain and the ribosome peptidyl transferase centre (PTC) to induce stalling, while the other key residues function to stabilise this interaction through positioning of the nascent chain within the exit tunnel [13]. The secondary interactions between SecM and the exit tunnel were shown by cryo-EM to include key interactions in the upper tunnel with 23S rRNA nucleotides A2062, U2585 and U2609 and in the mid-tunnel with A751 [14]. Indeed, insertion of an additional adenine residue within the five consecutive adenine residues A749–A753 abolished SecM stalling, highlighting that this area plays a function in nascent chain positioning [7]. The path of the SecM nascent chain through the ribosome exit tunnel, and its position in relation to selected ribosomal proteins and rRNA residues has been shown by Gumbart et al. (2012) [13].

The structure of the SecM nascent chain within the exit tunnel is critical to stalling as previous work has shown that compaction of the nascent chain within the exit tunnel is essential for translation arrest [15]. Formation of secondary structure within the ribosome is relevant to translation arrest as studies have revealed that some stalling peptides, such as *E. coli* SecM and CGS1 in *Arabidopsis thaliana*, undergo compaction in the exit tunnel upon translation arrest [15,16]. This compaction acts to position key residues within the exit tunnel allowing interactions to take place between the nascent chain and the tunnel walls, thus enabling stalling signals to be transmitted to the PTC. Previous FRET assays have revealed that the SecM peptide undergoes compaction at the C-terminus upon translation arrest and that this compaction is essential for stalling. The arrest occurs through a series of reciprocal interactions between the nascent chain and the exit tunnel, which are triggered by the addition of Pro^166^ to the PTC A-site, and functions to correctly position the key arrest residues to enable translation stalling [15]. MDFF modelling has further refined this initial discovery to reveal that this compaction occurs between residues W155 and R163, shortening the distance between the two residues from the ∼31 Å that would be expected if the peptide was in a fully extended conformation to 24 Å. This compaction was shown to locate and stabilise R163 in the vicinity of the 23S nucleotide A2062, the residue responsible for communicating stalling signals to the PTC [13]. It was also demonstrated that this movement of the nascent chain and its ability to adapt and interact with the exit tunnel is mediated by the amino acid sequence of the nascent chain as mutation of non-essential C-terminus residues S157, Q158 and Q160 to proline prevented compaction, which in turn, abolished stalling. Proline residues play an important role in protein structure as they restrict the flexibility, for a nascent chain. This, therefore, affects its movement and interactions within the exit tunnel [17,18]. A particular proline residue is explored in this study, Pro^153^ is a non-essential residue, which upon stalling and compaction of the nascent chain is situated at a key site within the exit tunnel, close to the constriction point 20–35 Å from the PTC [14]. While the cyclic structure of the proline side chain creates a rigid conformation, mutation to alanine, which has a small methyl side group (–CH_3_), increases the flexibility of the nascent chain. Woolhead et al. (2006) [14] demonstrated that while wild-type SecM could not undergo translation arrest in mutant ribosomes possessing expanded exit tunnels (Δ82–84 L22), incorporation of the P153A mutation recovered arrest of SecM peptides. The restoration of key interactions between the nascent chain and the ribosome exit tunnel provided by the removal of the restrictive proline residue re-enabled compaction of the nascent chain and subsequent translational stalling. Further to this, while it has been shown that SecM150–166 encompasses the essential arrest motif, when truncated constructs are translated, SecM140–166 was shown to be slightly more efficient at stalling than the shorter form [7]. This suggests that the residues further away from the C-terminus, while not forming essential interactions with the exit tunnel still play an important role in positioning the residues further up the nascent chain closer to the PTC, in particular in residue Arg^163^. Therefore, it remained to be explored whether increased freedom of movement out with the essential arrest motif, through mutation of non-essential Pro^146^ to alanine could also influence positioning of the key residues at the C-terminus of the SecM nascent chain.

The present study investigates the sequence specificity of the SecM arrest motif, in particular what properties of these amino acids are essential for their interaction with the exit tunnel to enable their role in positioning Arg^163^. To do this, the essential arrest residues, originally identified through alanine scanning mutagenesis [7] were individually mutated to conservative amino acids to determine what affect this has on translation arrest. In order to study the influence of nascent chain flexibility on stalling, double mutants containing alteration of a non-essential proline residues to alanine at positions within (P153A) and out with (P146A) the key arrest motif were created and analysed. Further analysis of the conservative SecM mutants by pegylation assays was used to explore the effect of these mutations on the compaction of the SecM nascent chain and the influence of the P153A mutation. In particular, how increased freedom of movement of the nascent chain relates to compaction and the resulting levels of stalling of these SecM mutants. Taken together, these results illustrate the significance of each individual arrest motif residue and their interactions with the exit tunnel that contribute to translation arrest.

## Experimental procedures

### Plasmid construction

pTrc-SecM was created as described by Woolhead et al. (2006) [14] and all further mutagenesis performed using QuickChange Site-Directed Mutagenesis kit (Agilent) with forward and reverse primers designed according to manufacturer’s instructions to incorporate the desired mutation.

### S-30 bacterial cell extract preparation

Bacterial strains C41 [19], CAW1 (C41 zhd33::tetrplV281) and CAW2 (C41 zhd33::tet rplD282) were used to make S-30 extracts essentially as described (Lesley et al. (1991) [20]). Five millilitres of overnight cultures were used to inoculate 500 ml of SOC medium (2% (w/v) tryptone, 0.5% (w/v) yeast extract, 0.05% (w/v) NaCl, 10 mM MgCl_2_, 10 mM MgSO_4_, 20 mM glucose) and incubated at 37°C in a shaking incubator to reach mid-log phase, A_600_ = 0.8. Cultures were then added to 1 l of ice and centrifuged at 4°C (7000 rpm, 10 min). The pellet was twice re-suspended in 100 ml of 20 mM HEPES (pH 7.5), 14 mM MgOAc, 100 mM KCl, 6 mM β-mercaptoethanol, 0.5 mM PMSF and centrifuged at 4°C (7000 rpm, 10 min). Cells were then re-suspended in 20 mM HEPES (pH 7.5), 14 mM MgOAc, 100 mM KCl, 1 mM DTT, 0.5 mM PMSF at a concentration of 0.5 g/ml. Lysozyme was then added to a final concentration of 1 mg/ml and the cells passed twice through a French Press at 8000 psi. The extract was centrifuged twice at 4°C (15000 rpm, 30 min) and the resulting supernatant incubated at 26°C for 70 min with 0.15 volumes of 0.75 mM HEPES (pH 7.5), 7.5 mM DTT, 21.3 mM MgOAc, 75 μM each amino acid, 6 mM ATP, 20 mg/ml phosphoenol pyruvate, 0.14 mg/ml pyruvate kinase. The extract was then dialysed three times in 20 mM HEPES (pH 7.5), 14 mM MgOAc, 100 mM KOAc, 1 mM DTT, 0.5 mM PMSF, then centrifuged at 4°C (14000 rpm, 10 min) and stored at −80°C.

### *In vitro* transcription translation reactions and cetyltrimethylammonium bromide precipitations

Linear DNA fragments were amplified using the ExTaq PCR kit (Takara) and the appropriate plasmid template. Standard 25 μl translation reactions were set up containing 0.5 μg linear DNA, 10 μl premix (Lesley et al. (1991) [20]), 2.5 μl 1 mM each l-amino acid (except methionine), 7.5 μl S-30 extract, 10 μCi [^35^S] methionine, 1 μl of 5 μg/μl anti-ssrA oligonucleotide (5′-TTAAGCTGCTAAAGCGTAGTTTTCGTCGTTTGCGACTA-3′). Reactions were incubated at 37°C for 30 min, then chilled on ice for 5 min and then mixed with 10 volumes of 2% (w/v) Cetyltrimethylammonium bromide (CTABr) and 10 volumes of 0.5M NaOAc (pH 4.7) and incubated on ice for a further 15 min before being centrifuged at room temperature (13400 rpm, 10 min). CTABr pellets were washed with 500 μl cold acetone. CTABr supernatant was incubated with 10% TCA on ice for 10 min before being centrifuged at 4°C (14000 rpm, 10 min) and TCA pellets were then washed with 1 ml cold acetone. All pellets were then centrifuged at 4°C (14000 rpm, 10 min). All samples were re-suspended in sample buffer and analysed by SDS/PAGE.

### Pegylation assays

Fifty microlitres of *in vitro* transcription–translation reactions were carried out with volumes altered accordingly. Translation reactions were chilled on ice for 5 min then overlaid on to a 100 μl sucrose cushion (0.5 M sucrose, 20 mM HEPES (pH 7.5), 14 mM MgOAc, 100 mM KOAc) and centrifuged at 4°C (100000 rpm, 6 min) using a Beckmann TLA-100 rotor. Pellets were re-suspended on ice in 20 mM HEPES (pH 7.2), 100 mM NaCl, 5 mM MgCl_2_ and divided in half. To one was added equal volume of 20 mM HEPES (pH 7.2), 100 mM NaCl, 5 mM MgCl_2_, 2 mM methoxy-polyethylene glycol maleimide (PEG-mal) (final PEG-mal concentration: 1 mM), while to the control was added equal volume of 20 mM HEPES (pH 7.2), 100 mM NaCl, 5 mM MgCl_2_. These were incubated on ice for 2 h before addition of 100 mM DTT and incubation for 10 min. Samples were then CTABr precipitated by addition of 10 volumes 0.5 M NaOAc (pH 4.7) and 10 volumes of 2% CTABr and incubated on ice for 15 min. They were then centrifuged at room temperature (13400 rpm, 15 min) and the pellet was re-suspended in 15 μl of 1 mg/ml RNaseA in ddH_2_O, followed by incubation at room temperature for 10 min. Samples were re-suspended in sample buffer and analysed by SDS/PAGE.

### ImageJ analysis

SecM arrest was quantified by ImageJ software and calculated as a percentage of [Arrested/(Arrested + Total full length)], with total full length being the sum of the full length protein in the pellet and supernatant. Each value is adjusted for background, and then taken as a proportion of wild-type SecM. The percentage pegylation was calculated as a percentage of [pegylated/(unpegylated + pegylated)] adjusted for background. Where average percentage values were shown these were calculated from *n*=3.

## Results

### Sequence specificity of the SecM arrest motif

**Figure 1:**
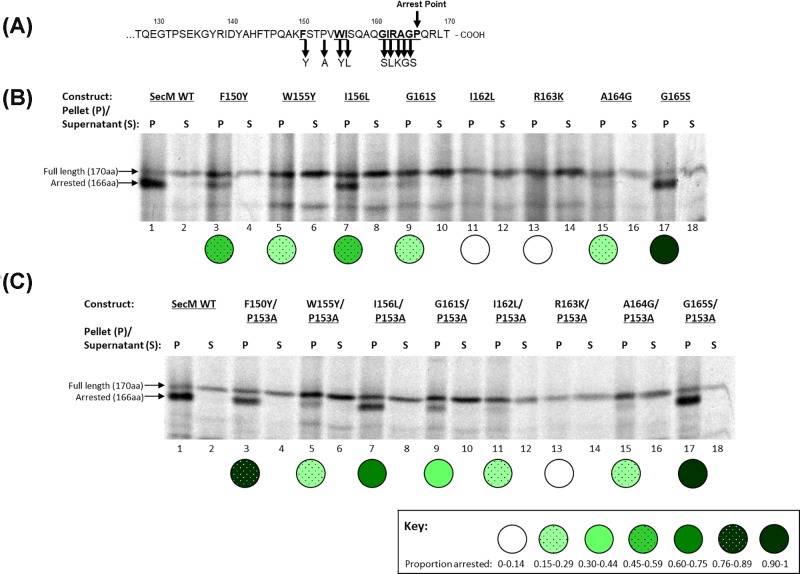
Stalling of single and double SecM conservative mutants (**A**) Schematic diagram of the mutations made to individual SecM constructs. (**B**) Single and (**C**) Double SecM conservative mutants were translated *in vitro* and CTABr precipitated, separated into pellet (P) and supernatant (S) fractions and resolved by SDS/PAGE.

The SecM stalling sequence was examined through analysis of SecM mutants by CTABr precipitation, CTABr binds to and precipitates RNA and by association, RNA–protein complexes. Therefore, in these experiments CTABr precipitates SecM that is stalled on the ribosome, due to its covalent attachment to the tRNA-Glycine [21]. The translation and CTABr precipitation of SecM indicates that the majority of the translation product is in the CTABr pellet fraction, this represents the arrested SecM as it migrated further on an SDS/PAGE gel than the full-length SecM, of which there is a small amount present in both the pellet and supernatant fractions (Figure 1B, lanes 1,2).

In 2002, Nakatogawa and Ito [7] carried out alanine scanning mutagenesis of the SecM arrest sequence in an *in vivo* experimental system. Using site-directed mutagenesis, the same mutations were introduced and tested in our *in vitro* system and the results, shown in Supplementary Figure S1, confirm the importance of the essential arrest motif residues, as only G165A was able to maintain any degree of stalling. This residue is located in the ribosome P-site upon stalling and therefore, unlike the other residues, which are located within the exit tunnel, may be able to accommodate the minor glycine to alanine mutation more effectively while still being an essential residue for efficient stalling. Supplementary Figure S1 also includes the results for the non-essential arrest motif residue P153 and confirms that when mutated to alanine, this residue maintains levels of stalling similar to the wild-type protein, as was initially shown by Woolhead et al. (2006) [14].

The key properties of the essential arrest motif amino acids were investigated by individually mutating these residues to conservative amino acids (Figure 1A), to identify whether the size or properties of the amino acids were critical to their involvement in translation arrest. The results indicate that G165S retains levels of stalling analogous to wild-type SecM (Figure 1B; lane 17), indicating that the properties of a serine residue can substitute for glycine and despite having a larger side chain, this is not detrimental to stalling. F150Y and I156L (Figure 1B, lanes 3,7) maintained levels of stalling of approximately 50% to that of wild-type SecM (Figure 1B, lane 1). Mutation of these residues to alanine abolishes stalling indicating that the structure of these side chains is important for their function. W155Y, G161S and A164G stalling is approximately 20% to that of wild-type SecM (Figure 1B, lanes 5,9,15 respectively). While I162L and R163K have the lowest stalling capability (Figure 1B; lanes 11,13 respectively), which is equivalent to that of their respective alanine mutants (Supplementary Figure S1B; lanes 13,15 respectively). These results support previous work that identified R163 as the key residue for translation arrest (Yap and Bernstein (2009) [12]); in addition these results show that the residues surrounding R163 cannot tolerate mutation, even to conservative amino acids, as efficiently as residues further away.

### Analysis of the effect of increased flexibility of the SecM nascent chain on the sequence specificity of the arrest motif

The P153A mutation was introduced into the single alanine mutant constructs, to create double mutants to examine the effect of an increase in the freedom of movement of the SecM nascent chain to accommodate the alteration of essential residues. With the exception of R163A and G165A, increased flexibility of the nascent chain allowed alanine mutations of the essential arrest motif residues to be better accommodated and restore some degree of stalling (Supplementary Figure S1C). Incorporation of the P153A mutation into single conservative mutant constructs has no further effect on the level of stalling for the residues G165S, A164G and R163K at the SecM C-terminus upon stalling (Figure 1C, lanes 13–18.), however, the remainder of the conservative mutations of the arrest motif residues had an increase in arrest with the P153A double mutation (Figure 1C, lanes 3–-12). As can be seen in the schematic diagram in Figure 1A, these residues are spaced apart on the nascent chain and not necessarily close to the P153A mutation, indicating that, away from the essential R163 residue, increased flexibility in the lower region of the arrest motif enables adaption and accommodation of conservative amino acid mutations within the ribosome exit tunnel.

**Figure 2:**
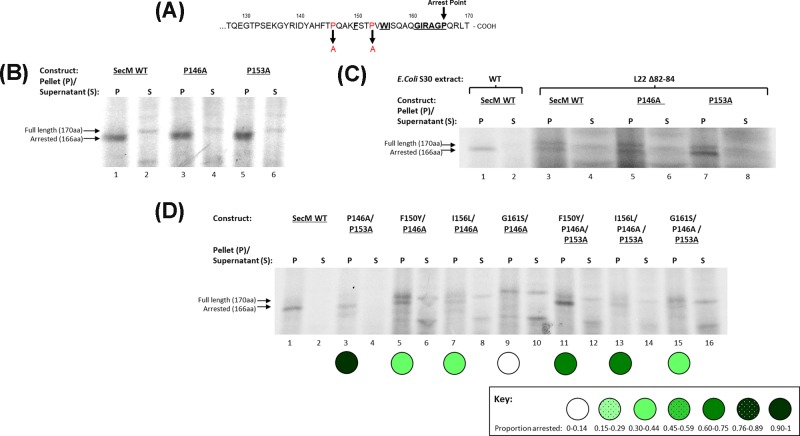
Effect of increased flexibility of SecM nascent chain within and out with the arrest motif on translation arrest (**A**) Schematic diagram indicating the position of the P146A and P153A mutations (highlighted in red) introduced into SecM constructs. The key arrest residues are highlighted in bold and underlined and the arrest point, Pro^166^, is indicated by an arrow. (**B**) SecM WT (lanes 1,2), P146A (lanes 3,4) and P153A (lanes 5,6) were translated *in vitro* and CTABr precipitated, separated into pellet (P) and supernatant (S) fractions and resolved by SDS/PAGE. (**C**) The constructs indicated were translated in coupled *in vitro* transcription–translation assays containing wild-type *E. coli* S30 cell extract (lanes 1,2) or cell extract derived from *E. coli* strain containing ribosomal deletion mutations of residues 82–84 of the L22 protein (lanes 3–8), which results in an expanded exit tunnel. The reactions were precipitated by CTABr, separated into pellet (P) and supernatant (S) fractions and resolved by SDS/PAGE. (**D**) SecM WT, P146A/P153A double mutant, double conservative mutations coupled with P146A and triple conservative mutations coupled with both P146A and P153A mutations were translated *in vitro* and CTABr precipitated, separated into pellet (P) and supernatant (S) fractions and resolved by SDS/PAGE.

An increase in flexibility of the SecM nascent chain out with the arrest motif was examined by mutating Pro^146^ to Alanine, a residue situated four amino acids downstream from the last residue of the arrest sequence, Phe^150^, and according to the study of Bhushan et al. (2011) [14], is located beyond the exit tunnel constriction site upon compaction and stalling (Figure 2A). CTABr precipitation assays show that, like the P153A mutation, the proline at position 146 can be mutated to alanine with no effect on the translation arrest efficiency of SecM (Figure 2B, lane 3). However, when translated using a ribosome extract derived from cells harbouring a deletion of residues 82–84 in the conserved β-hairpin of the L22 protein, a mutation that results in ribosomes with expanded exit tunnels has no significant effect on translation [22,23]. However, SecM translational arrest is prevented in these ribosomes (Figure 2C, lane 3) but the P153A mutation is able to restore stalling capability (Figure 2C, lane 7) (as previously seen in Woolhead et al. (2006) [14]), while SecM P146A (Figure 2C, lane 5), like w/t SecM, is also unable to stall the peptide. This suggests that an increase in flexibility further from the C-terminus and out with the arrest motif is unable to sufficiently influence the positioning of the key residues within it to influence stalling. When combined with selected single conservative mutations, P146A provided no arrest restoration, instead causing a reduction in levels of translation arrest (Figure 2D, lanes 5–10). Addition of P153A in a triple mutation (Figure 2D; lanes 11–16) resulted in levels of stalling that were comparable with that of the P153A double mutation (Figure 1C) indicating that P153A can still compensate and rescue stalling. As a control, a double mutant containing P146A/P153A was also created and tested and, as with the separate proline to alanine mutations, the level of stalling was analogous to wild-type SecM (Figure 2D, lane 3).

These results establish the importance of increased flexibility of the nascent chain within the SecM arrest motif in allowing the arrest essential amino acid residues to re-position and interact with the exit tunnel and in turn compensate for the mutation of key residues. An increase in flexibility further from the C-terminus and out with the arrest motif is unable to sufficiently influence the positioning of the residues within it.

### Analysis of SecM stalling and compaction by cysteine pegylation

**Figure 3:**
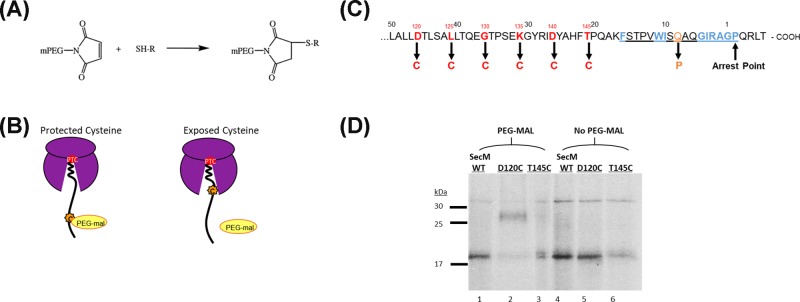
Pegylation as an assay for measuring nascent chain compaction within the ribosome exit tunnel (**A**) Structure of the PEG-mal functional group that covalently binds to the thiol group of cysteine. (**B**) Diagrammatic representation illustrating the protection of cysteine residues located within the ribosome exit tunnel upon stalling in comparison with the pegylation of exposed cysteine residues located outside the ribosome exit tunnel. (**C**) The sequence of the C-terminus of the SecM nascent chain highlighting the residues individually mutated to cysteine in red; Q158P in orange; and the arrest motif underlined, with the essential residues highlighted in blue. (**D**) SecM WT, D120C and T145C were translated *in vitro* and stalled ribosome–nascent chain complexes were isolated by ultracentrifugation through a 0.5 M sucrose cushion, divided in two and then incubated with 1 mM PEG-mal (lanes 1–3) or without (lanes 4–6). Pegylation of cysteine residues is indicated by a mass shift (1) of ∼10 kDa in comparison with unpegylated translation product (2).

The initial discovery of SecM compaction upon translation arrest was made by Woolhead et al. (2006) [14] and to further explore this we analysed the compaction of three conservative SecM mutants: F150Y, I156L and G161S; both individually and in tandem with the additional P153A mutation. This was done using a pegylation assay, a technique modified from that previously described by Lu and Deutsch (2005a&b) that employs PEG-mal, molecular weight 5 kDa, which binds covalently to the thiol groups of cysteine residues and results in an increase in the apparent molecular weight of the protein when separated on an SDS gel, see Figure 3A,D. These assays involve performing coupled *in vitro* transcription–translation assays, containing radiolabelled methionine, to produce stalled ribosome–nascent chain complexes with exposed cysteine residues of the stalled nascent chains then mass tagged with PEG-mal and detected by gel shift assay. PEG-mal is too large a molecule to enter the ribosome exit tunnel and therefore only cysteine residues on stalled nascent chains that are located outside the ribosome exit tunnel will be exposed to pegylation [24], see Figure 3B. As SecM contains no native cysteine residues, it can be selectively mutated to include single cysteine residues that are specifically located near the end of the ribosome exit tunnel when the peptide stalls, see Figure 3C.

The SecM residues selected for mutation to cysteine, D120 to T145, were selected as they cross the opening of the exit tunnel upon translation arrest (Figure 3B,C). Initial experiments confirmed that the exposed D120C residues were stably tagged with PEG-mal and resulted in a clear mass shift when the products were separated by SDS/PAGE (Figure 3D, lane 2). However, if there was no cysteine present, as in wild-type SecM (Figure 3D, lane 1) or the cysteine residue was still protected inside the ribosome exit tunnel, as in SecM T145C (Figure 3D, lane 3), then no pegylation occurred and there was no mass shift. An additional control was performed to determine the degree of cysteine pegylation achieved in the absence of the ribosome and associated factors, these experiments show that even though cysteines positioned at different places within the tunnel undergo differing degrees of pegylation, when released from the PTC by RNasA these values become normalised at 42.6–46.1% (Supplementary Figure S2, lanes 10,12).

**Figure 4:**
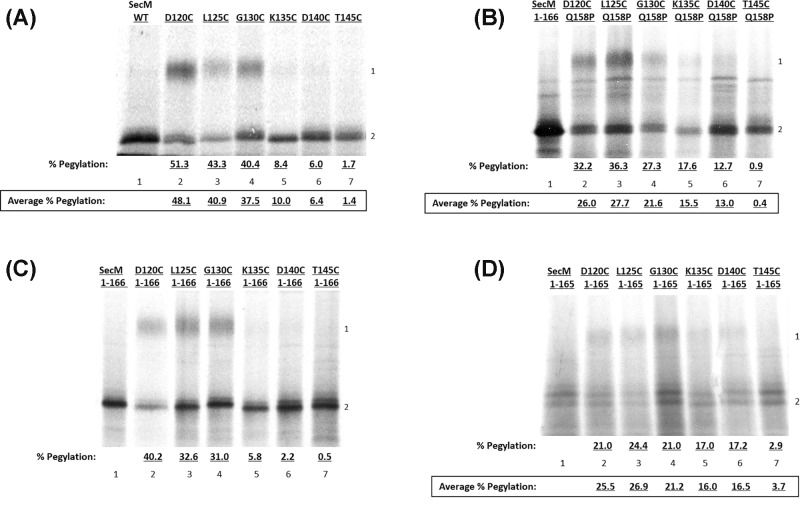
Pegylation assays of wild-type SecM and SecM Q158P (**A**) Wild-type SecM, (**B**) SecM Q158P, (**C**) SecM 1-166 and (**D**) SecM 1-165 constructs containing single cysteine mutations were translated *in vitro* and incubated with 1 mM PEG-mal before being CTABr precipitated and resolved by SDS/PAGE. Pegylation of cysteine residues is indicated by a mass shift of ∼10 kDa (1) in comparison with unpegylated translation product (2).

To define the point at which the stalled SecM nascent chain is exposed from the ribosome exit tunnel, the degree of pegylation across multiple points was determined in individual SecM cysteine mutants (D120C-T145C) (Figure 4A). Hypothetically, an extended nascent chain has 3.5 Å per amino acid and would therefore requires ∼28 residues to traverse the 100 Å length of the ribosome tunnel, while a complete α helical nascent chain has 1.5 Å per amino acid and would therefore require ∼67 residues to traverse the ribosome exit tunnel [25]. However, previous studies have shown that nascent chains traversing the exit tunnel do not form complete α helices throughout its full length, with certain parts of the exit tunnel more favourable to secondary structure formation than others [15,25,26]. Therefore, it is hypothesised that a partially α helical chain with 10 amino acids forming an α helix, with the remainder fully extended, would require ∼34 residues to traverse the exit tunnel, while a partially α helical chain with 20 amino acids in the helix with the remainder fully extended would require ∼40 residues to traverse the exit tunnel. The previous studies indicated that compaction occurred within defined areas of the exit tunnel however the pegylation assay does not discriminate between a tight compaction in one area of the nascent chain and an overall looser compaction of the full nascent chain throughout the length of the exit tunnel.

Wild-type SecM does not undergo pegylation as it does not contain any native cysteine residues to bind PEG-mal (Figure 4A, lane 1), while SecM cysteine mutants D120C, L125C and G130C undergo pegylation of ∼40–50% (Figure 4A; lanes 2–4 respectively). There is a dramatic reduction in pegylation at K135C to 8.4% and this continues to decrease the nearer the cysteine residue that is situated at the C-terminus, with 6.0% pegylation at D140C and only 1.7% at T145C (Figure 4A, lanes 5–7 respectively). These results indicate that residues K135C, D140C and T145C are protected from pegylation, indicating that 31–35 residues are still contained within the ribosome exit tunnel upon stalling and compaction of the SecM nascent chain. As mentioned previously, this is the equivalent to a partially α helical chain with 10 amino acids forming in an α helix with the remainder fully extended.

To enable comparisons to be drawn between compacted and extended SecM nascent chains, the SecM Q158P mutation was utilised. Residue Q158 is a non-essential amino acid located within the arrest motif and situated close to the essential R163 residue (see Figure 3C). While not an essential amino acid for arrest, it has been shown to interact with the 23S rRNA nucleotide A752 [13]. Due to the restrictive nature of proline, mutation in this residue prevents compaction and results in a loss of translation stalling [15]. As this peptide no longer undergoes translation arrest, to obtain stalled ribosome–nascent chain complexes in the *in vitro* transcription–translation assays it was necessary to use truncated linear DNA lacking a stop codon and containing Pro^166^ as the terminal residue. In the absence of a stop codon, mRNA remains associated with the ribosome, as there is no signal to initiate termination, allowing stalled ribosome–nascent chain complexes to be obtained.

These results indicate that the extended SecM Q158P undergoes pegylation at residues D120C–D140C (Figure 4B, lanes 2–6), while T145C remains unpegylated (Figure 4B, lane 7), indicating only residue T145C remains within the ribosome exit tunnel upon stalling. This indicates that between 21 and 25 residues protected by the exit tunnel, a difference of ∼10 amino acids from compacted WT SecM. The overall percentage of pegylation is reduced in comparison with wild-type SecM with the highest average level of pegylation of WT SecM being 48.1% at D120C (Figure 4A, lane 2), compared with 26.0% in SecM Q158P (Figure 4B, lane 3). This is most likely due to differences in the secondary or tertiary structure of the nascent chain outside the exit tunnel, which in the case of SecM Q158P results in the cysteine residue being more protected and therefore consequently decreasing the accessibility of PEG-mal, resulting in a reduced overall rate of pegylation. Despite this greater protection of the cysteine residue there is still a clear segregation in the level of pegylation between residues outside of the exit tunnel and those protected within it, as pegylation of SecM Q158P at residue T145C reduces to only 0.9% (Figure 4B, lane 7).

The compaction of truncated SecM constructs has previously been assayed through FRET analysis by Woolhead et al. (2006) [15], in which they established that SecM does not undergo compaction until the full arrest motif including Pro^166^ has been synthesised. Truncated SecM 1-166 and 1-165 single cysteine constructs were utilised to examine the pegylation rates of extended (1-165) and compacted (1-166) SecM stalled on the ribosome. The pegylation of SecM 1-166 is analogous to wild-type SecM, with these results indicating that the percentage pegylation of SecM 1-166 is between 40.2 and 31.0% at residues D120C, L125C and G130C (Figure 4C, lanes 2–4 respectively), with a significant decrease to 5.8% pegylation at K135C (Figure 4C, lane 5). This further decreases to 2.2 and 0.5% at D140C and T145C respectively (Figure 4C, lanes 6,7 respectively). These results again indicate that when SecM compacts upon stalling the nascent chain crosses the opening of the exit tunnel between residues G130C and K135C. In contrast, the extended SecM peptide is protected within the exit tunnel only at residue T145C, with only 2.9% of the peptide undergoing pegylation (Figure 4D, lane 7), and the other peptides, D120C–D140C undergoing between 24.4 and 17.2% pegylation (Figure 4D, lanes 2–6 respectively). These results support the previous data in Figure 4A,B that compacted SecM has between 31 and 35 residues contained within the exit tunnel upon stalling and compaction, while extended SecM has between 21 and 25 residues.

### Influence of individual SecM mutations on compaction of the nascent chain upon stalling

**Figure 5:**
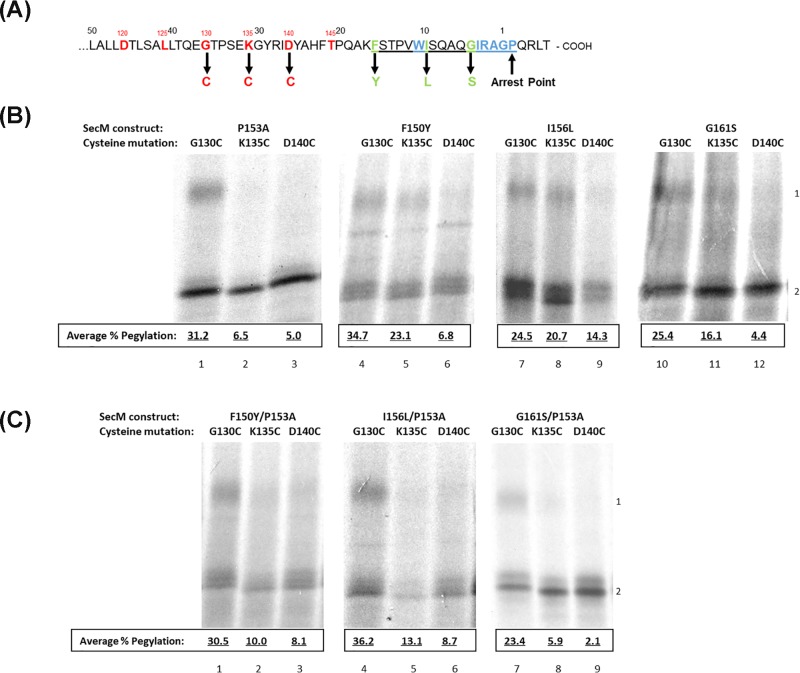
Pegylation results of conservative SecM mutants (**A**) Schematic diagram of the C-terminus of SecM, the conservative mutations made to separate SecM constructs are highlighted in green with the arrest motif underlined and the remaining unmodified essential residues shown in blue. Additional mutations were made to these constructs as described in the text including: G130C, K135C and D140C mutations which are shown in red. (**B**) Pegylation results for SecM P153A (lanes 1–3), SecM F150Y (lanes 4–6), SecM I156L (lanes 7–9) and SecM G161S (lanes 10–12) single cysteine constructs G130C, K135C and D140C. (**C**) Pegylation results for SecM F150Y/P153A (lanes 1–3), SecM I156l/P153A (lanes 4–6) and SecM G161S/P153A (lanes 7–9) single cysteine constructs G130C, K135C and D140C. These peptides were translated *in vitro* as previously described, divided in half, with one half as control and the other incubated with 1 mM PEG-mal before being CTABr precipitated and resolved by SDS/PAGE. Pegylation of cysteine residues is indicated by a mass shift of ∼10 kDa (1) in comparison with unpegylated translation product (2).

The compaction of SecM P153A was analysed by pegylation and the results are shown in Figure 5B. Constructs containing cysteines at three sites were focused on: G130C, K135C and D140C, the schematic diagram of these constructs can be seen in Figure 5A. The results indicate that the P153A mutation has no effect on nascent chain compaction upon stalling in comparison with wild-type SecM. G130C undergoes 31.2% pegylation (Figure 5B, lane 1) which reduces to 6.5 and 5.0% at K135C and D140C respectively (Figure 5B, lanes 2,3) indicating that, like wild-type SecM, the nascent chain compacts and protects residues K135C and D140C within the ribosome exit tunnel. Following this, the compaction and stalling of three separate conservative SecM mutations: F150Y, I156L and G161S were assayed. While the average pegylation rates of SecM F150Y residues G130C (34.7%) and D140C (6.8%) (Figure 5B, lanes 4,6) remain relatively similar to that of wild-type SecM (Average = 37.5 and 6.4% respectively) (Figure 4A, lanes 4,6), the increased pegylation of SecM F150Y at residue K135C (Average = 23.1%) (Figure 5B, lane 5) in comparison with wild-type (Average = 10%) (Figure 4A, lane 5) indicates that this peptide adopts a less compact structure within the exit tunnel upon stalling, resulting in increased exposure of K135C from the exit tunnel and hence increased pegylation. These results indicate that 26–30 residues of SecM F150Y are contained within the ribosome exit tunnel upon stalling, in comparison with 21–25 residues of fully extended SecM. This suggests that there is still partial folding or helix formation but not as much as wild-type SecM (31–35 residues contained within the exit tunnel). SecM G161S undergoes a similar degree of compaction as SecM F150Y with residue D140C, 4.4% pegylation (Figure 5B, lane 12), remaining protected by the ribosome exit tunnel with increased pegylation of K135C (16.1%) indicating increased exposure of the residue from the exit tunnel (Figure 5B, lane 11).

Of the three conservative mutants, I156L adopts the most extended conformation upon stalling, comparable with a fully extended SecM peptide (Figure 5B, lanes 7–9) with pegylation present at all three cysteine residues (G130C–D140C: Average 24.5, 20.7 and 14.3% respectively) indicating all three residues are exposed and only 21–25 residues remain within the exit tunnel. Also, as seen with the extended peptides, SecM Q158P and truncated SecM 1-165, the overall rates of pegylation are lower than wild-type SecM, with SecM I156L having a highest average pegylation at G130C of 24.5% (Figure 5B, lane 7) in comparison with wild-type SecM which has 40.4% (Figure 4A, lane 4). This indicates that the SecM I156L peptide also undergoes secondary or tertiary structure outside the exit tunnel similar to the other extended peptides SecM Q158P and SecM 1-165, reducing access of PEG-mal to the cysteine residues of the extended peptide.

### Investigating the effect on compaction of additional flexibility of the nascent chain resulting from P153A mutation

The effect of increased flexibility of the nascent chain provided by the P153A mutation on the compaction of the SecM nascent chain was explored by creating double mutants of the previously selected conservative mutants: F150Y, I156L and G161S. The results of the pegylation assay of the SecM F150Y/P153A double mutant are shown in Figure 5C and indicate that incorporation of the P153A mutation results in increased compaction of the nascent chain. While G130C pegylation remains high at an average of 30.5% indicating it is positioned outside the exit tunnel and exposed to PEG-mal binding (Figure 5C, lane 1), average pegylation of K135C decreases from 23.1% in SecM F150Y (Figure 5B, lane 5) to an average of 10.0% when combined with the P153A mutation (Figure 5C, lane 2). Meanwhile D140C remains protected by the exit tunnel and undergoes an average of 8.1% pegylation (Figure 5C, lane 3). This indicates that compaction of the nascent chain is restored when combined with the P153A mutation, indicated by the withdrawal of the K135C residue further into the exit tunnel protecting it from PEG-mal binding.

**Figure 6:**
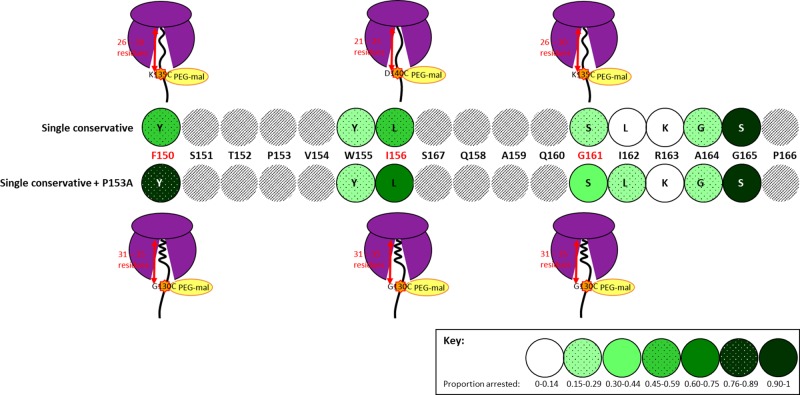
Combined summary of translation arrest and degree of compaction for the selected conservative SecM mutants studied Highlighted in red are the three conservative SecM mutations which were investigated in the pegylation assay (F150, I156 and G161) to determine number of residues contained within the ribosome exit tunnel, and therefore the degree of compaction of the SecM nascent chain upon translation arrest.

Incorporation of the P153A mutation to SecM I156L restores compaction of the SecM nascent chain with pegylation of the exposed G130C residue of the SecM I156L/P153A double mutant increased to an average of 36.2% (Figure 5C, lane 4) and K135C and D140C reduced to 13.1 and 8.7% respectively (Figure 5C, lanes 5,6 respectively) indicating that these residues are now protected within the exit tunnel upon stalling and compaction. Finally, SecM G161S/P153A double mutant show that G130C had an average of 23.4% pegylation (Figure 5C, lane 7) while K135C and D140C reduced to an average of 5.9 and 2.1% respectively (Figure 5C, lanes 8,9 respectively). This decrease in pegylation indicates that residues K135C and D140C are protected within the ribosome exit tunnel. These results show that for all three conservative mutants studied, while the single mutant had a more extended conformation upon stalling with only 26–30 residues contained within the exit tunnel, in combination with the P153A double mutation, partially α helix structure of the nascent chain similar to WT SecM was again restored, with 21–25 residues contained within the exit tunnel upon stalling and compaction, summarised in Figure 6.

## Discussion

### SecM partially withstands single conservative mutations to key arrest residues

From this work it has been established that the ability of the SecM peptide to tolerate mutation of key arrest residues to conservative amino acids is dependent on their location within the exit tunnel upon inducing arrest. Arg^163^ is a key residue in the SecM peptide and cannot tolerate alteration, even a conservative mutation to lysine results in loss of the SecM stalling ability. Interaction between arginine and the ribosome exit tunnel does not appear to be transient as it is highly specific. Instead it suggests that the arginine residue may bind within a specific rRNA pocket and interact with rRNA nucleotide A2062, to induce structural changes that signal to PTC, leading to the ratcheting of the tRNA moiety and ultimately translational stalling [14]. Having such a highly specific location and amino acid requirement is critical as this reduces the chances of similar peptides wrongly inducing translation arrest.

In addition, residue W155 is also intolerant to alteration in the SecM peptide resulting in loss of stalling ability due to the interactions of the W155 residue within the exit tunnel. While both W155 and I156 have been shown to make contact with the exit tunnel at rRNA nucleotide A751 [14], despite the close proximity of these residues to each other in the nascent chain it is the tryptophan that is the more essential residue (Figure 1B). This correlates with previous data which have shown that a tryptophan residue 11–12 amino acids from the PTC is a key feature which is conserved among some stalling peptides including SecM such as AAP and TnaC [7,27,28].

The properties of specific amino acids also have varying importance depending on where they are positioned within the SecM nascent chain, again the closer to the essential R163 the less tolerance there is for alteration. For instance, I162, which is located beside the critical R163 and may also have subsidiary interactions with nucleotide A2062 [14], does not tolerate conservative mutation to lysine and cannot be greatly compensated for by an increase in flexibility at P153A (Figures 1B,C, both lane 11). However further down the nascent chain I156L has relatively high levels of stalling for the same amino acid modification (Figures 1B,C, both lane 7). Likewise, glycine to serine, when located at residue 165 at the ribosome P-site upon stalling, has far greater scope for modification than the same mutation at position 161 which is located within the exit tunnel (Figures 1B,C, lanes 9,17 respectively). In the case of G161 the amino acid may be responsible for interacting with the tunnel wall or positioning the nascent chain within the tunnel while the role of Gly^165^, which is located in the P-site of the ribosome upon stalling, is mediated through the movement of the tRNA-Gly in preventing peptide bond formation upon translation arrest [14], and not the properties of the amino acid itself. Of the key arrest motif residues, G165 is able to withstand mutation most effectively and can be mutated to serine without impacting stalling. There is genetic evidence for a serine residue at position 165 in SecM variants of other species, including *Mannheimia succiniciproducens*, which may account for its ability to maintain wild-type levels of stalling in *E. coli* SecM [29].

### Increased flexibility of the SecM nascent chain by P153A mutation enables repositioning of key residue, Arg^163^

Increased flexibility in the lower region of the arrest motif, below Arg^163^, provided by the P153A mutation, enables repositioning of conservative SecM amino acid mutants within the ribosome exit tunnel to compensate for the mutations and enable increased levels of stalling (Figure 1C, lanes 3–12). The residue furthest from R163 has the greatest scope for alteration, with F150Y the most accommodating of the conservative mutations with ∼80% arrest with the P153A double mutation (Figure 1C, lane 3).

The increase in flexibility of the nascent chain provided by the P153A mutation does not, however, appear to correct above Arg^163^ as increased flexibility at P153A has no effect on the stalling capability at A164G and G165S mutants (Figure 1C, lanes 15–18). The importance of G165 is in the shift the tRNA undertakes which prevents the formation a peptide bond with Pro^166^ upon stalling, this appears to be due to alterations within the PTC and cannot be optimised by the positioning of the nascent chain within the exit tunnel. A164 is the last residue to be located in the exit tunnel upon stalling and despite both alanine (–CH_3_) and glycine (–H) having small side chains, the methyl group of the alanine residue must still have an important role in translation arrest and the communication of the stalling signal to the PTC. Previous studies have shown that residues R163-G165 form critical interactions during translation arrest [30], with these results supporting the importance of the unique interactions made within the exit tunnel while those within the PTC can withstand mutation.

To influence the translation arrest capability of the SecM mutants the increase in flexibility of the nascent chain must come from within the essential arrest motif, as increased movement provided by mutation of another proline residue out with the essential motif, at P146, had no ability to recover stalling in SecM mutants (Figure 2D). The P146 residue is located beyond the ribosome exit tunnel constriction point [14], which appears to null any ability to influence SecM nascent chain positioning closer to the PTC. An increase in flexibility of the nascent chain is only beneficial in the region within the arrest motif that is located in the upper tunnel upon arrest.

These results have shown that a combination of both nascent chain flexibility within the arrest motif, and amino acid specificity are required for SecM stalling. The SecM nascent chain is highly dynamic within the exit tunnel and is able accommodate mutations of essential residues to varying degrees. Some residues are more important than others, in particular those located close to, and including, the essential R163 residue. Out with R163, this work highlights that it is not necessarily how an individual residue behaves on its own, but how the C-terminus of SecM as a whole combines to position the key R163 residue. The ability of the P153A mutation to recover stalling of some mutants indicates that these SecM peptides must position themselves differently within the tunnel to allow the more effective communication of the stalling signal to the reach the PTC.

### Cysteine pegylation as a method to analyse SecM stalling and compaction within the ribosome exit tunnel

Cysteine modification by PEG-maleimide is a technique that has previously been exploited to detect nascent peptide conformation within the ribosome exit tunnel and this technique has been utilised here to assay the level of SecM compaction upon translation arrest. The pegylation assay enabled the full degree of compaction within the whole length of the ribosome exit tunnel to be measured, and from this, comparisons are to be drawn between wild-type SecM and several SecM mutants.

Hypothetically, an extended nascent chain has 3.5 Å per amino acid and would therefore require ∼28 residues to traverse the 100 Å length of the ribosome tunnel, while a complete α helical nascent chain has 1.5 Å per amino acid and would therefore require ∼67 residues to traverse the ribosome exit tunnel [25]. However previous studies have shown that nascent chains traversing the exit tunnel do not form complete α helices throughout its full length, with certain parts of the exit tunnel more favourable to secondary structure formation than others [15,25,26]. Therefore, it is hypothesised that a partially α helical chain with 10 amino acids forming an α helix, with the remainder fully extended, would require ∼34 residues to traverse the exit tunnel, while a partially α helical chain with 20 amino acids in the helix with the remainder fully extended would require ∼40 residues to traverse the exit tunnel. The previous studies indicated that compaction occurred within defined areas of the exit tunnel, however, the pegylation assay does not discriminate between a tight compaction in one area of the nascent chain and an overall looser compaction of the full nascent chain throughout the length of the exit tunnel.

These results indicate that wild-type SecM and truncated SecM 1-166 have between 31 and 35 residues contained within the exit tunnel upon stalling and compaction (Figures 4A,C respectively), while the extended SecM Q158P and SecM 1-165 peptides have between 21 and 25 residues protected by the exit tunnel, a difference of ∼10 amino acids (Figure 4B,D). These values are close to the hypothesised values which estimate that a fully extended peptide would require ∼28 residues to traverse the exit tunnel while a partially α helical chain would take ∼34–40 residues and are representative of a partially α helix compaction of the SecM nascent chain upon stalling. While these results illustrate that pegylation is never 100% efficient, this is possibly due to SecM nascent chain out with the ribosome exit tunnel during stalling has been shown to have an important role in efficient SecM stalling [31], which would in turn result in a shielding of the cysteine residue from complete PEG-mal binding.

### Influence of single conservative mutations on compaction of SecM nascent chain

The structure of the SecM nascent chain within the exit tunnel appears to be dynamic and mutations of key arrest motif residues can be compensated for by increased freedom of movement of the nascent chain, allowing re-positioning of the nascent chain upon stalling and thereby enabling accommodation of mutations of key amino acid residues. The question that remained was what happened to the compaction of the SecM nascent chain during this re-positioning to accommodate these mutations. The results indicate that of the three conservative SecM mutants studied, F150Y and G161S underwent only slight compaction upon stalling (26–30 residues contained within the ribosome exit tunnel upon stalling), not equivalent to the partially α helical structure of wild-type SecM (31–35 residues contained within the exit tunnel), whilse SecM I156L remained in an extended conformation (21–25 residues within the exit tunnel), see Figure 5B. Despite this loss of compaction SecM F150Y and I156L maintain stalling capability of ∼55% while SecM G161S is ∼25%, see Figure 6 for a summary of these results.

The main interaction between the SecM nascent chain and the ribosome exit tunnel that is responsible for translation arrest occurs at rRNA nucleotide A2062, however there are other interactions between the nascent chain and exit tunnel which occur near the L4/L22 constriction point which are also important but appear less essential [13]. In particular in this region residue W155 of SecM has been shown to base stack with nucleotide A751 of 23S rRNA. As I156L is directly besides W155 and this SecM mutant remains in an extended conformation, this suggests that mutation of this residue to leucine prevents base stacking between W155 and A751, resulting in the extended conformation of SecM I156L upon stalling. The loss of base stacking at A751 due to the proximity of the I156L mutation would explain why F150Y and G161S mutations, which are further away, affect compaction to a lesser degree. Despite this the level of extension or compaction, does not correlate with the level of stalling, as SecM I156L has the highest level of translation arrest out of the three conservative mutations studied. This suggests that compaction of the SecM nascent chain does not directly determine the stalling capability of the peptide, instead stalling appears to be based on a more refined ability of the nascent chain to position the key R163 residue correctly within the ribosome exit tunnel. For wild-type SecM this is achieved through compaction of the nascent chain, however these results show that SecM mutants can be accommodated by alternative positioning and interactions of the nascent chain within the exit tunnel. In turn, this accounts for the high level of variation in the sequence and behaviour of other stalling peptides which do not rely on nascent chain compaction to achieve translation arrest, such as AAP [32].

### Influence of additional flexibility of the nascent chain resulting from P153A mutation on compaction of SecM mutants

Mutation of P153A alone does not affect the efficiency of SecM stalling in comparison with wild-type (Supplementary Figure S1, lane 5), neither does it have any effect on the overall compaction of SecM upon stalling (Figure 5B, lanes 1–3). However, when combined with single conservative mutations it resulted in a return of nascent chain compaction to levels analogous to that of wild-type SecM, for all three conservative mutations studied (Figure 5C). This also correlated with an increase in translation arrest however, unlike nascent chain compaction, the levels of translation arrest do not return to that of wild-type SecM, see Figure 6 for an overall summary. Although not possible to conclude from these experiments, the close proximity of P153A to residue W155 may account for this occurrence. MDFF modelling revealed that compaction of the SecM nascent chain upon stalling occurs between residues W155 and R163, and results in a shortening of the distance between these residues from ∼31 Å in a fully extended conformation to 24 Å [13]. The increased flexibility of the SecM nascent chain provided by the P153A mutation adjacent to the base stacking of W155 with rRNA nucleotide A751 appears to enable accommodation of the conservative mutations and return compaction of the nascent chain to that of wild-type SecM levels. However, although the results indicate that the increased flexibility of the SecM nascent chain restores the levels of compaction to that of wild-type SecM, it is unable to completely compensate for the loss of the essential amino acid residues, despite their mutation to closely related conservative amino acids. The behaviour of these mutants to restore compaction when provided with increased flexibility reinforces again that compaction of SecM upon stalling provides the optimum positioning of R163 hence why, with increased flexibility, these peptides return to the compacted structure upon stalling.

In summary, the increase in the levels of translation arrest of the studied conservative SecM mutants by the P153A double mutation is due to the increased freedom of movement of the nascent chain within the exit tunnel, which enables the repositioning of key amino acid arrest residues. The resulting increase in translation arrest indicates that the interactions between the nascent chain and the ribosome exit tunnel are a key mediator in the level of translation arrest. However, although the additional P153A mutation increases the level of stalling of the F150Y, I156L and G161S mutants, these results indicate that recovery of full compaction of the SecM nascent chain does not result in a full recovery of translation arrest, see Figure 6 for an overall summary of the results. Taken together these results have shown that compaction of SecM is not reliant on the detection of a completely accurate arrest motif, however, complete stalling capability is.

## Supporting information

**Supplementary Figures:** 

## Abbreviations

CTABr: cetyltrimethylammonium bromide
MDFF: molecular dynamics flexible fitting
PEG-mal: methoxy-polyethylene glycol maleimide
PTC: peptidyl transferase centre
SecM: secretion monitor

## References

[1] Sarker S., Rudd K.E. and Oliver D. (2000) Revised translation start site for secM defines an atypical signal peptide that regulates *Escherichia coli* secA expression. J. Bacteriol. 182, 5592–5595 10.1128/JB.182.19.5592-5595.2000 10986266 PMC111006

[2] Evans M.S., Ugrinov K.G., Frese M.A. and Clark P.L. (2005) Homogeneous stalled ribosome nascent chain complexes produced *in vivo* or *in vitro*. Nat. Methods 2, 757–762 10.1038/nmeth790 16179922

[3] Cabrita L.D., Hsu S.-T.D., Launay H., Dobson C.M. and Christodoulou J. (2009) Probing ribosome-nascent chain complexes produced in vivo by NMR spectroscopy. Proc. Natl. Acad. Sci. U.S.A. 106, 22239–22244 10.1073/pnas.0903750106 20018739 PMC2799734

[4] Jomaa A., Boehringer D., Leibundgut M. and Ban N. (2016) Structures of the E. coli translating ribosome with SRP and its receptor and with the translocon. Nat. Commun., 7, 1–7 10.1038/ncomms10471 PMC4737761 26804923

[5] Nakatogawa H. and Ito K. (2001) Secretion monitor, secM, undergoes self-translation arrest in the cytosol. Mol. Cell 7, 185–192 10.1016/S1097-2765(01)00166-6 11172723

[6] Nakatogawa H., Murakami A., Mori H. and Ito K. (2005) SecM facilitates translocase function of SecA by localizing its biosynthesis. Genes Dev. 19, 436–444 10.1101/gad.1259505 15713839 PMC548944

[7] Nakatogawa H. and Ito K. (2002) The ribosomal exit tunnel functions as a discriminating gate. Cell 108, 629–636 10.1016/S0092-8674(02)00649-9 11893334

[8] McNicholas P., Salavati R. and Oliver D. (1997) Dual regulation of *Escherichia coli* secA translation by distinct upstream elements. J. Mol. Biol. 265, 128–141 10.1006/jmbi.1996.0723 9020977

[9] Butkus M.E., Prundeanu L.B. and Oliver D.B. (2003) Translocon “pulling” of nascent SecM controls the duration of its translational pause and secretion-responsive secA regulation. J. Bacteriol. 185, 6719–22 14594848 10.1128/JB.185.22.6719-6722.2003 PMC262105

[10] Goldman D.H., Kaiser C.M., Milin A., Righini M., Tinoco I. and Bustamante C. (2015) Ribosome. Mechanical force releases nascent chain-mediated ribosome arrest in vitro and in vivo. Science 348, 457–60 10.1126/science.1261909 25908824 PMC4618485

[11] Muto H., Nakatogawa H. and Ito K. (2006) Genetically encoded but nonpolypeptide prolyl-tRNA functions in the A site for SecM-mediated ribosomal stall. Mol. Cell 22, 545–552 10.1016/j.molcel.2006.03.033 16713584

[12] Yap M.N. and Bernstein H.D. (2009) The plasticity of a translation arrest motif yields insights into nascent polypeptide recognition inside the ribosome tunnel. Mol. Cell 34, 201–11 10.1016/j.molcel.2009.04.002 19394297 PMC2704006

[13] Gumbart J., Schreiner E., Wilson D.N., Beckmann R. and Schulten K. (2012) Mechanisms of SecM-mediated stalling in the ribosome. Biophys. J 103, 331–341 10.1016/j.bpj.2012.06.005 22853911 PMC3400775

[14] Bhushan S., Hoffmann T., Seidelt B., Frauenfeld J., Mielke T., Berninghausen O. (2011) SecM-stalled ribosomes adopt an altered geometry at the peptidyl transferase center. PLoS Biol. 9, 10.1371/journal.pbio.1000581 21267063 PMC3022528

[15] Woolhead C.A., Johnson A.E. and Bernstein H.D. (2006) Translation arrest requires two-way communication between a nascent polypeptide and the ribosome. Mol. Cell 22, 587–598 10.1016/j.molcel.2006.05.021 16762832

[16] Onoue N., Yamashita Y., Nagao N., Goto D.B., Onouchi H. and Naito S. (2011) S-adenosyl-L-methionine induces compaction of nascent peptide chain inside the ribosomal exit tunnel upon translation arrest in the *Arabidopsis* CGS1 gene. J. Biol. Chem. 286, 14903–14912 10.1074/jbc.M110.211656 21335553 PMC3083191

[17] Chou P.Y. and Fasman G.D. (1974) Conformational parameters for amino acids in helical, beta-sheet, and random coil regions calculated from proteins. Biochemistry 13, 211–222 4358939 10.1021/bi00699a001

[18] O’Neil K.T. and DeGrado W.F. (1990) A thermodynamic scale for the helix-forming tendencies of the commonly occurring amino acids. Science 250, 646 10.1126/science.2237415 2237415

[19] Miroux B. and Walker J.E. (1996) Over-production of protein in *Escherichia coli*: mutant hosts that allow synthesis of some membrane proteins and globular proteins at high levels. J. Mol. Biol. 260, 280–289 10.1006/jmbi.1996.0399 8757792

[20] Lesley S.A., Brow M.A. and Burgess R.R. (1991) Use of in vitro protein synthesis from polymerase chain reaction-generated templates to study interaction of Escherichia coli transcription factors with core RNA polymerase and for epitope mapping of monoclonal antibodies. J. Biol. Chem. 266, 2632–8 1703532

[21] Gilmore R., Collins P., Johnson J., Kellaris K. and Rapiejko P. (1991) Transcription of full-length and truncated mRNA transcripts to study protein translocation across the endoplasmic reticulum. Methods Cell Biol. 34, 223–239 10.1016/S0091-679X(08)61683-0 1943802

[22] Chitum H.S. and Champney W.S. (1994) Ribosomal protein gene sequence changes in erythromycin- resistant mutants of *Escherichia coli*. J. Bacteriol. 176, 6192–6198 7928988 10.1128/jb.176.20.6192-6198.1994 PMC196958

[23] Gabashvili I.S., Gregory S.T., Valle M., Grassucci R., Worbs M., Wahl M.C. (2001) The polypeptide tunnel system in the ribosome and its gating in erythromycin resistance mutants of L4 and L22. Mol. Cell, 8, 181–188 10.1016/S1097-2765(01)00293-3 11511371

[24] Lu J. and Deutsch C. (2001) Pegylation: a method for assessing topological accessibilities in Kv 1.3. Biochemistry 40, 13288–13301 10.1021/bi0107647 11683639

[25] Lu J. and Deutsch C. (2005) Folding zones inside the ribosomal exit tunnel. Nat. Struct. Mol. Biol., 12, 1123–1129 10.1038/nsmb1021 16299515

[26] Bhushan S., Hoffmann T., Seidelt B., Frauenfeld J., Mielke T., Berninghausen O. (2011) SecM-stalled ribosomes adopt an altered geometry at the peptidyl transferase center. PLoS Biol. 9, 1–10 10.1371/journal.pbio.1000581 PMC3022528 21267063

[27] Freitag M., Dighde N. and Sachs M.S. (1996) A UV-induced mutation in neurospora that affects translational regulation in response to arginine. Genetics 142, 117–127 8770589 10.1093/genetics/142.1.117 PMC1206940

[28] Gong F. and Yanofsky C. (2002) Instruction of translating ribosome by nascent peptide. Science 297, 1864–1867 10.1126/science.1073997 12228716

[29] Yap M.N. and Bernstein H.D. (2011) The translational regulatory function of SecM requires the precise timing of membrane targeting. Mol. Microbiol. 81, 540–553 10.1111/j.1365-2958.2011.07713.x 21635582 PMC3134173

[30] Zhang J., Pan X., Yan K., Sun S., Gao N. and Sui S.F. (2015) Mechanisms of ribosome stalling by SecM at multiple elongation steps. Elife 4, e09684 10.7554/eLife.09684 26670735 PMC4737659

[31] Yang Z., Iizuka R. and Funatsu T. (2015) Nascent SecM chain outside the ribosome reinforces translation arrest. PLoS ONE, 10, 1–13 10.1371/journal.pone.0122017 PMC4373844 25806953

[32] Wu C., Wei J., Lin P.J., Tu L., Deutsch C., Johnson A.E. (2012) Arginine changes the conformation of the arginine attenuator peptide relative to the ribosome tunnel. J. Mol. Biol. 416, 518–533 10.1016/j.jmb.2011.12.064 22244852 PMC3568992

