# Supplementary figures and images for: Increased freedom of movement in the nascent chain results in dynamic changes in the structure of the SecM arrest motif
Source: Biosci Rep. 2019 Jan 18;39(1):BSR20181246. doi: 10.1042/BSR20181246 (PMC6340945; doi:10.1042/BSR20181246)

A.

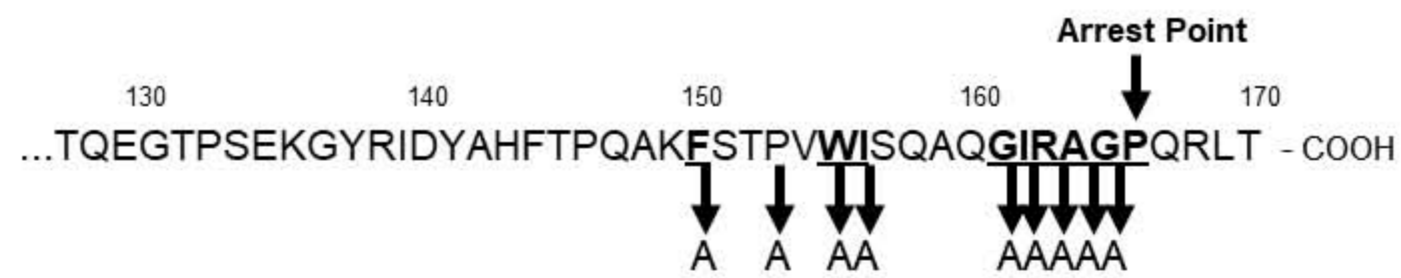

B.

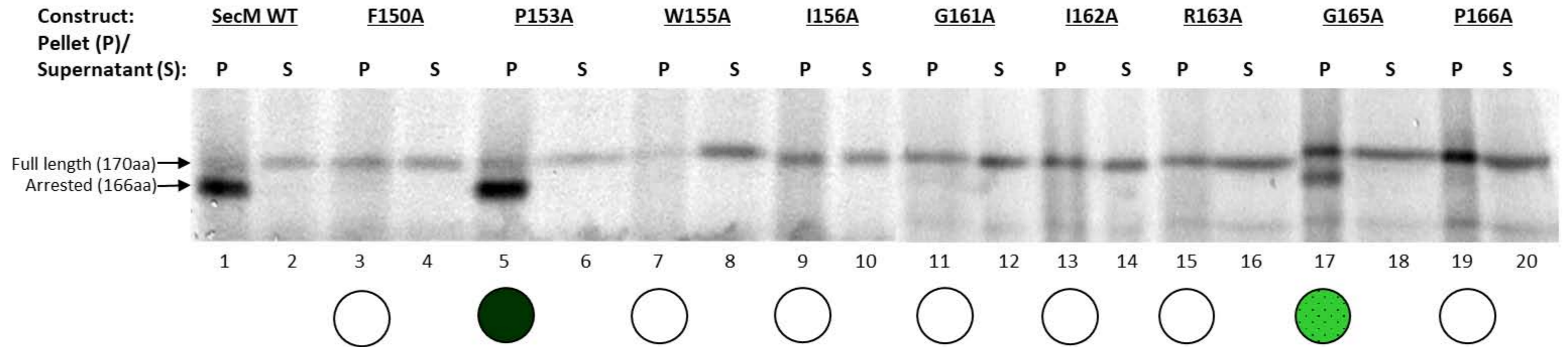

C.

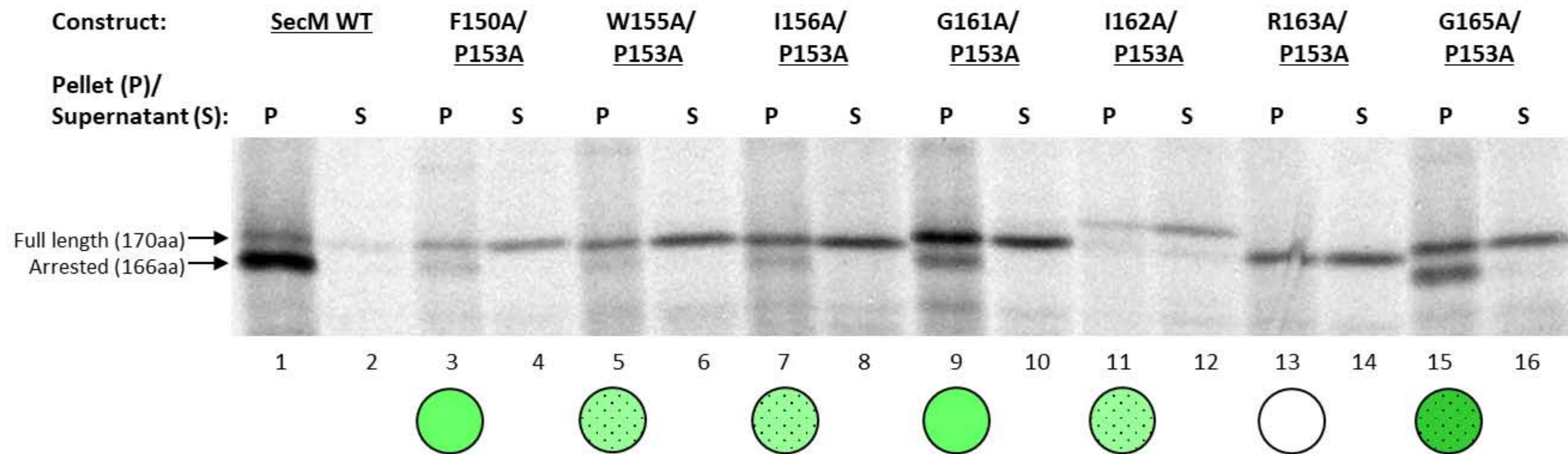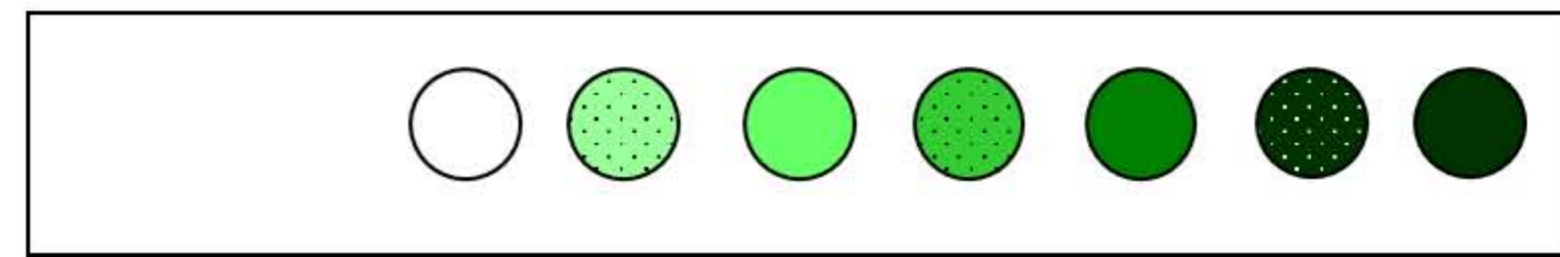

Supplementary Figure 1.

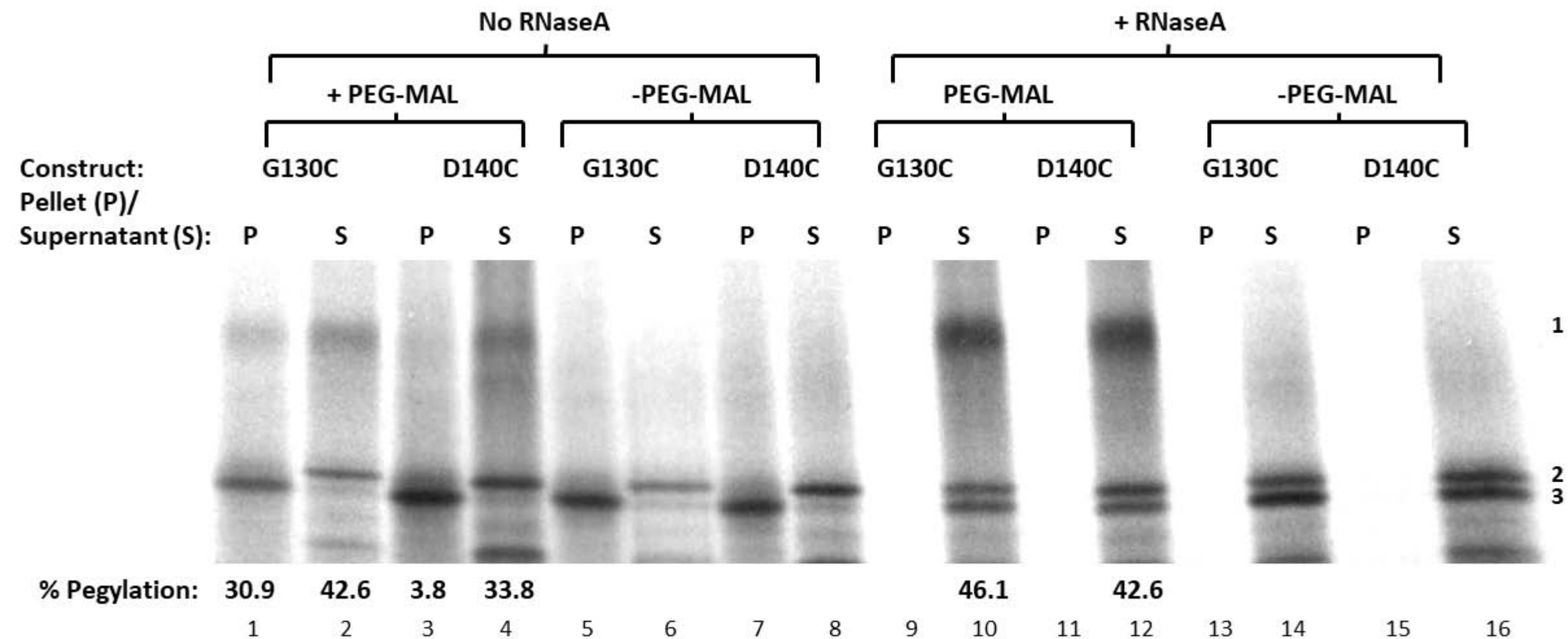

**Supplementary Figure 2.**

Supplement: Supplementary file 1 [file bsr20181246_Supp1.pdf]
